# How health care may modify the effects of illness determinants on population outcomes: the Leicester SEARCH conceptual framework for primary care

**DOI:** 10.3399/bjgpopen18X101603

**Published:** 2018-08-22

**Authors:** Louis S Levene, John Bankart, Nicola Walker, Andrew Wilson, Richard Baker

**Affiliations:** 1 Honorary Lecturer, Department of Health Sciences, College of Life Sciences, University of Leicester, George Davies Centre for Medicine, Leicester, UK; 2 Honorary Associate Professor in Medical Statistics, Department of Health Sciences, College of Life Sciences, University of Leicester, George Davies Centre for Medicine, Leicester, UK; 3 Research Fellow, Department of Health Sciences, College of Life Sciences, University of Leicester, George Davies Centre for Medicine, Leicester, UK; 4 Professor of Primary Care, Department of Health Sciences, College of Life Sciences, University of Leicester, George Davies Centre for Medicine, Leicester, UK; 5 Professor Emeritus, Department of Health Sciences, College of Life Sciences, University of Leicester, George Davies Centre for Medicine, Leicester, UK

**Keywords:** Primary Care, Epidemiology, Public Health

## Introduction

To research, evaluate, and deliver health care that effectively improves health outcomes across populations, relationships between the numerous variables that determine these outcomes should be understood. Conceptual frameworks can aid the description and analysis of health in populations. Investigators usually have an implicit framework underpinning their research. Population health lags behind other disciplines, such as psychology and sociology, in the use of conceptual frameworks;^1^ currently published frameworks are not configured ideally for primary care-focused research. In this article, we aim to fill an important gap by describing a new and comprehensive conceptual framework for population health that can assist both research and service in primary care. The framework provides a schematic overview of presumed relationships between variables, recognising that many variables do not ‘behave’ consistently in every situation.

### Population health: definitions and its role in improving health care

The World Health Organization (WHO) defines health as ‘a state of complete physical, mental and social well-being, and not merely the absence of disease or infirmity’.^2^ Disease is a disorder of structure or function, which produces specific symptoms or affects a specific location; it is not solely a direct result of trauma. Health needs are ‘deficiencies in health that require health care, from promotion to palliation’.^2^ Health outcomes are the end results or effects of events or processes or situations. Health care is the organised provision of medical care to individuals or a community,^3^ covering a range of interventions (actions taken to improve health). Healthcare systems are the combined activities of people, institutions, and resources whose primary purpose is to promote, restore, and/or maintain health.^3^ Illness determinants are the range of personal, social, economic, and environmental factors that decisively affect health status or cause illness.^3^

Healthcare systems can struggle to deliver better health outcomes for whole populations. Effective interventions are more likely to be delivered if the relevant underlying factors and their relationships within populations are recognised in areas such as illness determinants, health needs, and health-related outcomes, and the likely effects of interventions on these. Tackling the causes of important outcomes should be a priority, with systems being prepared to adopt long-term solutions. However, policy makers frequently require healthcare systems to undertake interventions for short-term gains or to focus simultaneously on multiple unprioritised targets and performance measures. A large multimethod study identified barriers to delivering high quality and safe care: these included unclear goals, overlapping priorities, and excessively bureaucratised management.^4^

### Conceptual frameworks in population health

Conceptual frameworks are analytical tools that aim to give an overview of the extent and major features of a system of connected characteristics and their inter-relatedness. Frameworks are used to make conceptual distinctions and organise ideas, and seek to represent reality in a simplified or schematic way.^5^ They may vary in scale (areas covered or complexity) and context (for example, applied science, social science, or economics); thus, their definitions and applications are likely to vary. The design of a conceptual framework should be governed by its purpose: this includes exploration (for example, clarifying aims, developing working hypotheses, and generating realistic and relevant research questions), description, analysis (for example, selecting appropriate methods and identifying potential validity threats), decision making (for example, operational), explanation (including justifying research), and prediction (for example, formal hypothesis specification).^6^ Population health frameworks may include variables relating to population characteristics, disease mechanisms, morbidities, and their effects on populations, outcomes, and interventions (for example, health care).

Previous conceptual frameworks in population health have varied in several ways, particularly with respect to:

1. Scope, with either a comprehensive perspective^7,8^ or focusing mainly on a specific area. These areas have included descriptions of illness,^9^ the synergistic interaction of two or more disease states producing a set of linked health-related problems (syndemics),^10,11^ population shifts of risk factors,^12^ primary care’s impact on population health^13,14^ and primary care organisation.^15–20^ A single condition, diabetes, is the subject of a useful framework that links socioeconomic status to different health outcomes in patients. These relationships can be modified by various ‘proximal mediators/moderators’, classified as knowledge and self-care health behaviours, access, or process of care.^21,22^
2. Configuration of components, which is usually determined by the framework’s purpose. Differentiation between direct or indirect effects of variables on other variables, or between structures and processes within hierarchies of variables, are not always described, even in some comprehensive frameworks.^9^
3. Intended users. Some frameworks are designed for researchers.^7,12^ Others are aimed at policy makers in healthcare systems (operational),^8,23^ and may have a primary care focus.^15–20^ Nevertheless, these frameworks may require adjustment to help general practices deliver care that improves population health outcomes.

Taking the above issues into account and incorporating useful elements from previous frameworks, we developed a new comprehensive framework. Its configuration has a general practice perspective and is designed to aid those researching and delivering health care that contributes to improved population health outcomes.

## Description

### Development

In our first empirical study, we explained two-thirds of the cross-sectional variation in all-age coronary heart disease (CHD) mortality between English primary care trusts (PCTs). Population variables, especially deprivation, were the main predictors, but detection of hypertension, a healthcare performance variable, was also a predictor.^24^ These findings led us to postulate a conceptual framework in which non-disease variables are important predictors of variations in population mortality rates. These predictive effects may be modified by health care, which includes improving access (for example, continuity of care) and risk factor detection (for example, hypertension). Better healthcare access and risk factor detection are two of Starfield’s six mechanisms for explaining the beneficial impact of primary care on population health.^13^ In subsequent studies (discussed in the next section), we used our framework to develop research questions and interpret results. Despite subsequent modifications, the framework’s postulations remain the same.

Primary care is more likely than specialist care to exploit the full extent of the framework, which is generalisable for any healthcare system. Primary care looks after whole populations, dealing usually with the entire span of the natural history of illnesses, addressing a broader range of health needs, and undertaking an extensive range of interventions.

### Overview

**Figure 1.**
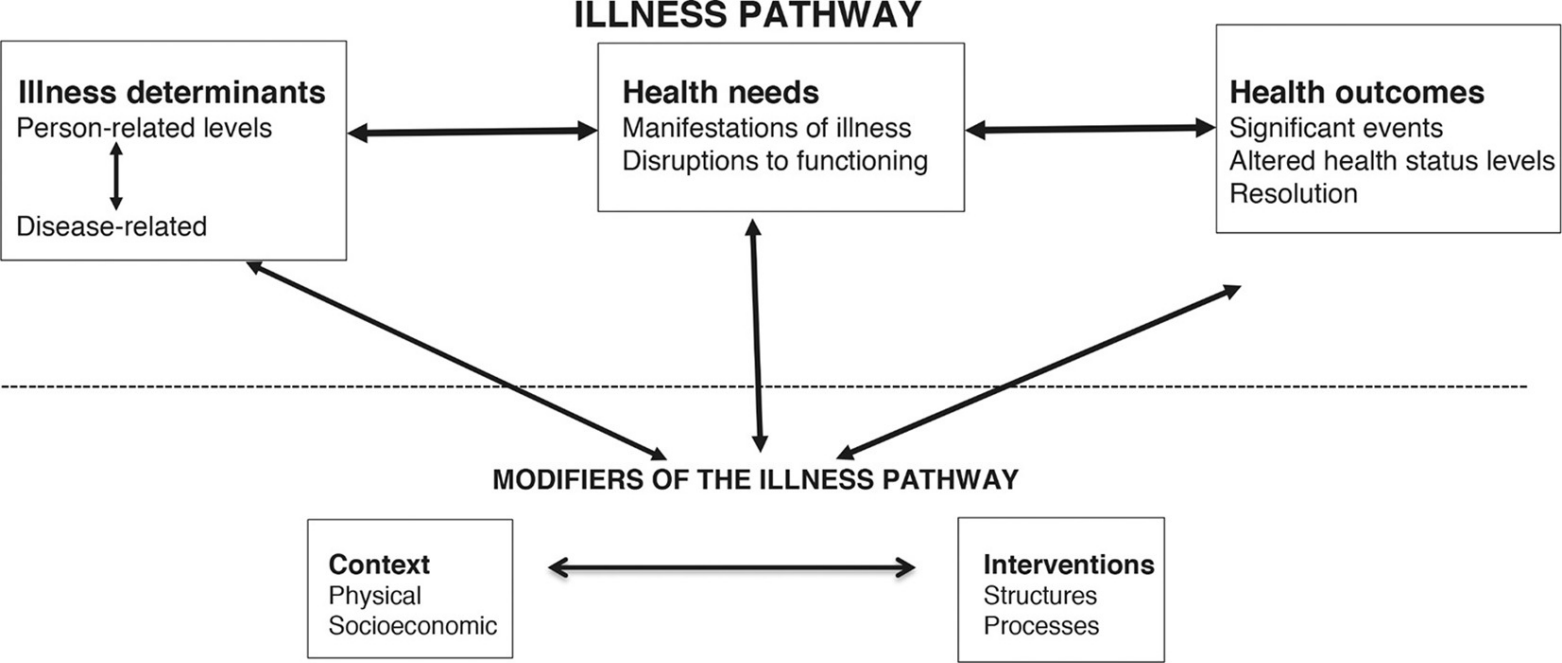
Configuration of the Leicester SEARCH (Systematic Exploration and Analysis of Relationships Connecting Health variables in populations) conceptual framework.

Our new framework, named the Leicester SEARCH (Systematic Exploration and Analysis of Relationships Connecting Health variables in populations) conceptual framework, has two components (Figure 1):

1. An illness pathway consisting of three groups of variables, starting with illness determinants (subdivided into person-related and disease-related variables), which generate health needs (subdivided into manifestations of illness and disruptions to functioning), that then predict health-related outcomes in populations.
2. Modifiers of the illness pathway, consisting of two groups of variables, context (factors that are not directly involved in the generation of illness but describe the settings in which populations are located) and interventions (for example, healthcare variables described as structures or processes). These variable groups may act either on modifiable illness determinants that generate health needs or on health needs themselves, thus influencing the onset of resulting health outcomes.

The following example is used to illustrate SEARCH. In the illness pathway:

- Tuberculosis (TB), an infectious disease, is usually caused by the bacterium *Mycobacterium tuberculosis*, with its level of activity influenced by the presence or absence of risk factors, for example, smoking and concurrent illnesses such as HIV/AIDS (illness determinants).
- In turn, these may influence the prevalence of both disease and clinical features (health needs —﻿ manifestations of illness), and the disease’s effects on individuals’ lives (health needs —﻿ disruptions to functioning).
- Subsequently, these may influence the onset of health-related outcomes, such as death, hospitalisation, or complications.

However, this illness pathway may be modified in different populations by:

- Context: TB is closely linked to adverse socioeconomic conditions with overcrowding and malnutrition, especially in resource-poor communities. Additionally, poverty may affect access to (including prevention and screening programmes) and compliance with health care (poor compliance is associated with the rise of drug resistance); thus, the context may not only affect illness determinants, health needs, and outcomes, but also interact with interventions.
- Interventions: structures, for example, policies and available resources, affect the settings and efficacy of TB healthcare delivery. Processes may include prevention (such as, infant vaccination), screening, diagnosis (such as, by clinical sample), and management (such as, drug regimens), including collaboration between different services.

### Variable groups

Variables should be precisely defined and, ideally, measurable. Their inclusion in the conceptual framework is dependent on their having health-related importance. Individual variables are allocated to one of the five main groups: illness determinants, health needs, health-related outcomes, context, and interventions (Box 1 and Figure 1). Each large group is subdivided into smaller groups.

**Box 1.** Detailed classification of variables within the Leicester SEARCH conceptual framework. These variables describe populations and may be quantified as prevalences or levels.

**A. Illness Pathway: Illness determinants**		
** Group**	**Subgroup**	**Examples**
Person-related	Population characteristics	Age, sex, and ethnicity structures
Person-related	Biological	Levels of raised blood pressure, obesity, adverse lipid profiles, genetic predispositions
Person-related	Behavioural	Patterns across populations in diet, physical activity, smoking behaviour, alcohol consumption, health literacy and self-sufficiency, illicit substance use, and engagement with systems
Disease-related	Disease mechanisms	Infections, external injuries, developmental abnormalities, genetic susceptibilities, autoimmune defects, and cellular degeneration
Disease-related	Epidemiology	Prevalence, natural history, and distribution in populations
**B. Illness Pathway: Health needs**		
**Group**	**Subgroup with examples**	
Manifestations of illness	1. Levels of morbidity (for example, disease prevalence in given populations) 2. Prevalence and types of clinical features (for example, physical or psychological)	
Disruptions to functioning (in those with disease)	1. Physical (for example, activities of daily living, such as mobility, feeding, continence, or dressing) 2. Psychological (for example, scores on tools measuring anxiety and depression, or dementia) 3. Socioeconomic (changes in roles, for example, job, relationships, and in activities, for example, driving, sports)	
**C. Illness Pathway: Health outcomes**		
**Group**	**Subgroup**	**Examples**
Significant events	Complication	1. Death (for example, disease-specific rates, rates by population characteristic) 2. Non-fatal (for example, rates of non-fatal myocardial infarction, diabetes-related limb loss, or exacerbation of chronic obstructive pulmonary disease)
Significant events	Transfer of care	1. Hospital admission rates (emergency or planned), 2. Referrals to secondary care (rates or types) 3. Discharge rates between units or into the community
Altered health status levels	Physical/psychological	Population rates of disability-adjusted life years
Altered health status levels	Socioeconomic	Rates of unemployment or of disability benefit claims
Resolution	Cure	Rates of disease eradication
Resolution	Remission	Rates of those with inactive disease, but not confirmed as eradicated
**D. Modifiers: Context**		
**Group**	**Subgroups with examples**	
Physical	1. Geography (for example, urban versus rural) 2. Housing (including homelessness) 3. Transport 4. Facilities 5. Water supply and sanitation 6. Exposure to crime 7. Exposure to pollution	
Socioeconomic	1. Public and private policies (for example, governmental at different levels) 2. Expenditure on social welfare and education 3. Social networks (relationships, families, carers, or other support) 4. Education (including linguistic and numerical literacy) 5. Employment 6. Fiscal (for example, income and expenditure)	
**E. Modifiers: Interventions**		
**Group**	**Subgroup**	**Specific areas with examples**
Structures	Governance	1. Policies at government, local, and organisational levels 2. Regulations: legal, organisational, and professional
Structures	Resources	1. Financial (expenditure, allocation, or remuneration/incentives) 2. Human (numbers, distribution and characteristics; for example, skill sets) 3. Material (for example, physical infrastructure or IT systems)
Processes	Access	1. Physical and organisational distribution (for example, optimising choice and availability of place of care between home, or community and institution) 2. Appointment systems (for example, flexibility or timing) 3. Candidacy (targeting best care to all those with the potential to benefit)
Processes	Range of work	1. Anticipatory care (prevention plus early detection) 2. Acute care 3. Management of long term conditions (for example, coronary heart disease, stroke, COPD, thyroid, or chronic severe mental illness)
Processes	Coordination	1. Teamwork 2. Pathways to other healthcare providers, other care (for example, social) networks, and with public health systems
Processes	Continuity and choice of care	1. Balance between long-term relationship with usual provider, if preferred, and freedom to choose provider 2. Management (access to effective IT systems with appointments, medical records, medical prescribing, communications within and outside, or financial/contractual data)

A variable can belong to more than one group, depending on its effects in different situations. Policies may belong to both the context and intervention groups. Variables relating to cultural and religious practices may be classified as context, with modifying effects on illness pathways in some populations; but in other populations their effects can be sufficiently powerful to act as illness determinants within illness pathways.

In the illness determinants group, variables are classified as either person-related or disease-related. Person-related variables describe the susceptibility of a group of individuals to develop illness. These can be further divided into population characteristics (often unmodifiable: for example, age or ethnicity profiles), biological (sometimes modifiable: for example, prevalence of genetic predispositions, raised blood pressure, abnormal lipid profiles, or obesity), and behavioural subgroups (often modifiable; for example, patterns of dietary habits, physical activity, smoking, or health literacy and self-sufficiency). Disease-related variables describe the illnesses affecting these populations and are divided into disease mechanisms and epidemiology.

Within the illness pathway, interactions between person-related and disease-related factors generate the next group of variables: the health needs of a population. These may be diverse^9^ and are divided into two subgroups:

1. manifestations of disease, which relate to the prevalence of morbidity and of clinical features (physical and psychological); and
2. disruptions to functioning (in those with disease), which can be physical, psychological, or socioeconomic. These relate to population levels of variables describing health status. Examples include mobility, self-care, usual activities, pain/discomfort, and anxiety/depression, which are assessed in the EuroQol Group’s EQ-5D questionnaire.^25^

Death may be certain,^26^ but its timing and that of other outcomes are linked to the presence and nature of illness determinants and health needs. In our framework, health outcomes variables are classified as significant events (for example, rates of deaths, non-fatal complications, and hospital admissions), altered health status levels (physical/psychological, for example, disability-adjusted life years, or socioeconomic, for example, rates of unemployment or benefit claims), or resolution (cure or remission). The rates of different outcomes in populations are determined by numerous factors, including disease mechanisms, population characteristics, and the efficacy of interventions (provision and modes of action).

The remaining two large groups of variables, context and interventions, are modifiers of populations’ trajectories along the illness pathway. Context includes a wide range of non-medical variables describing the settings in which populations are found. These variables’ behaviour and effects may vary between individuals, between places or within time. Occasionally the effects are sufficiently powerful to cause disease (thus, acting as illness determinants), but mostly the effects only influence trajectories of populations along the illness pathway. Context variables are classified as either physical or socioeconomic (hierarchies shown in Box 1). Calculations of relative socioeconomic deprivation, such as the Index of Multiple Deprivation (IMD), combine information from numerous variables. As many of these are context variables, IMD could fit into the framework as a compound context variable.

Interventions may be delivered in different settings (for example, community, hospitals, and other facilities) and be of different types (for example, primary health care, secondary healthcare, social care, and self-care). SEARCH classifies intervention variables as either structures or processes. Structure variables describe the organisation of healthcare systems and are further classified as either governance or resources. Process variables describe the range of actions undertaken by healthcare systems and are further classified as access, range of work, coordination, or continuity and choice of care (Box 1).

The effects and quality of interventions can be monitored by a range of methods that may examine process- or health-related outcomes.^27^ Variables used in evaluations may belong to different groups within SEARCH: interventions (for example, how providers of health care are organised or the success of mechanisms for delivering interventions), health needs (for example, changes in disease incidence), and outcomes (for example, mortality or other complication rates).

### Relationships between variables

The term ‘relationship’ refers here to the empirical association between two variables. SEARCH aims to provide a logical means of considering, describing, and analysing the huge range of presumed relationships between variables located either within the same group or in different groups (examples in Box 2). A causal association between two variables can be tested only through appropriately designed empirical studies, using appropriate statistical methods and guided by a conceptual framework based on existing scientific knowledge.

**Box 2.** Examples of relationships between variables within the Leicester SEARCH conceptual framework

Interaction type	Example
The risk of a Health Need (manifestation of illness) leading to a Health Outcome (mortality), modified by Interventions	Increased mortality rates if higher coronary heart disease (CHD) prevalence, but these rates can be reduced by smoking cessation, and by optimising blood pressure and lipid profiles in target populations.
Context (physical environment) affects the interaction between disease-related and person-related Illness Determinants to generate a Health Need	Air pollution combined with smoking increase the prevalence of chronic pulmonary disease and acute infections
Context modifying Illness Determinant leading to Health Need	UK legislation to ban smoking in buildings and other public places leading to fewer smokers and a reduction in age-related CHD mortality rates
Interaction between Context and Intervention	Influx of migrants with specific health needs may require health services to provide additional or different forms of health care to address their needs, with the aim of improving these populations’ health outcomes

Relationships between variables may be complex. If the degree of association between predictor **X1** and outcome **Y** depends on the level of a third variable **X2**, then there is an interaction between **X1** and **X2.** Confounding is present if predictor **X1** and predictor **X2** are both associated with outcome **Y**, and also with each other. A variable’s associations may not be consistent and may differ between populations. Such fluctuations can depend on whether the study is cross-sectional or occurs over time (longitudinal). Declines in CHD mortality rates have been associated with improved blood pressure detection and control, and with decreasing numbers of smokers. However, the future decline of these rates could lessen, halt, or reverse, due to being offset by rising prevalences of diabetes and obesity, or to slower changes in numbers of smokers and those with controlled blood pressure.

Within the illness determinants group, the epidemiology of illnesses is determined by combinations of person-related and disease-related variables whose underlying mechanisms may differ. The mechanisms of variables affecting CHD prevalence may be biological (for example, increasing age or genetic predisposition), behavioural (for example, smoking, diet, or physical activity), or a combination of both (for example, obesity, blood pressure, or lipid profiles); interactions between variables may also affect illness prevalence. Also, modifying variables may alter the effect of illness determinants on generating illness and on outcomes; for example, raised levels of air pollution and more smokers may amplify each other’s effects on both the prevalence and the consequences of chronic lung diseases (Box 2).

If a variable’s behaviour can be altered by intervention, then that variable is modifiable. Modifiable variables include many health-related behaviours and risk factors; for example, smoking, or blood pressure. Conversely, a non-modifiable variable’s behaviour cannot be altered by intervention; for example, age or ethnicity. The modifiability of variables should be considered when planning, implementing, and evaluating interventions. If interventions modify one or more variables and affect their relationships with other variables, then this may alter the onsets of health needs and of health-related outcomes (as predicted by health needs).

Trajectories along the illness pathway may be bi-directional: health-related outcomes may generate changes in health needs, or even in other outcomes. Higher rates of resolution or remission may reduce population health needs. Conversely, increased rates of diabetes complications (significant events), such as lower limb loss and visual impairment, could limit the types or extent of activities undertaken in affected populations or lead to changes in employment rates; for example, for some groups of drivers (health needs such as disruptions to functioning).

## Discussion

SEARCH has both research and service utility. It recognises the importance of prevention, where interventions, which modify risk factors in affected populations, may delay the onset and lessen the effects of illness. This focus is timely in the UK, as the *Five Year Forward View*,^28^ published in 2016, called for a significant and radical upgrade of prevention-related interventions delivered by the NHS.

### Research applications

The framework informed our selection of predictor and confounder variables, based on conceptual plausibility, and how we interpreted our findings in the following studies:

1. In four disease groups (CHD, stroke, all cancers, and chronic obstructive pulmonary disease [COPD]), there were large cross-sectional variations in all-age mortality between English PCTs, and, as in our first study, these variations were explained mostly by population variables.^29^
2. Premature (defined as <75 years of age) CHD mortality in England declined by two-thirds between 1993 and 2010. During this period, mortality declined more in PCTs with greater deprivation and more smokers, but relative variations in mortality between PCTs increased;^30^ a finding consistent with other studies.^31,32^
3. Population variables, in particular deprivation, were the most powerful predictors of cross-sectional variations between all English general practices in premature all-cause mortality; however, having more GPs per head of population was an independent predictor of lower premature mortality rates, even in a health system based on universally accessible primary care. In less deprived practices, greater continuity of care was associated with lower mortality.^33^
4. The weak cross-sectional association between individual general practices’ funding levels and levels of population factors that predict health needs suggests that English primary care may struggle to help reduce health inequalities.^34^
5. The decline over time of patient-perceived relationship continuity of care in English primary care is persistent and widespread. Deprivation scores did not predict variations in this decline; practice organisational and other population factors predicted weakly or not at all.^35^

### Service applications

Clinical commissioning groups and local authorities should collaborate, by developing clear and sensible priorities for local health, public health, and social care, while reflecting national policies. This requires agreement on what local outcomes are most important, and then on how best to tackle these outcomes’ causes. Examples include ensuring cost-effective hospital bed occupancy by reducing avoidable admissions and accelerating appropriate discharges, and taking a more proactive approach to screening and managing risk factors, such as obesity and hypertension, that contribute to increased morbidity and demands on services. A conceptual framework can be used to identify and organise relevant factors and their possible relationships, helping organisations to develop better policies and to manage interventions more effectively. Continuing evaluations can guide adjustments to providers’ structures and processes.

SEARCH can aid evaluations of cost-effectiveness, such as by deciding whether to implement interventions, after weighing up the resources required against their effectiveness in reducing health needs and improving outcomes. Such calculations should take into account relationships and interactions between variables, and variations in their levels and behaviours, especially between different population cohorts.

SEARCH is consistent with a theoretical approach which addresses population health needs by identifying and delivering interventions that:

- target not just disease mechanisms, but also important modifiable population characteristics (including those linked to socioeconomic circumstances);
- are readily accessible to those at greatest risk within populations;
- are delivered by services working collaboratively; and
- are prioritised by cost-effectiveness for populations, within the limits of available resources.

These are more likely to improve population health outcomes and to reduce health inequalities.

### Strengths and limitations

SEARCH was developed in parallel with a programme of research studies. It is compatible with other conceptual frameworks, but it represents an advance on them with respect to primary care use. It includes a large number of commonly described or measured variables and outlines possible relationships between variables in a way that reflects reality. SEARCH allows differentiation between variables’ direct and indirect effects on illness and outcomes, with sufficient flexibility to recognise variability in these effects, thus reducing the need to postulate numerous extra assumptions that are not specifically mentioned within the framework. SEARCH can aid the research, evaluation, and delivery of community-based health care that contributes to improved population health outcomes.

Limitations include the possible existence of unknown and, therefore, overlooked types of variables. These, if established, may require review of SEARCH’s configuration. An ideal balance needs to be found between sufficient simplicity for clarity and sufficient complexity to give a more comprehensive representation of reality: a risk in all frameworks. SEARCH has been formulated specifically to explain phenomena at population level rather than at individual level: ecological fallacies may occur when using population level data or frameworks to incorrectly infer the role of illness determinants on health needs and outcomes at individual level. However, as relationships between factors at both population and individual levels may have similarities, a conceptual framework designed for use at one level might thus be modified for use at another level.
